# Natural diversity of glycoside hydrolase family 48 exoglucanases: insights from structure

**DOI:** 10.1186/s13068-017-0951-5

**Published:** 2017-11-30

**Authors:** Roman Brunecky, Markus Alahuhta, Deanne W. Sammond, Qi Xu, Mo Chen, David B. Wilson, John W. Brady, Michael E. Himmel, Yannick J. Bomble, Vladimir V. Lunin

**Affiliations:** 1 Biosciences Center, National Renewable Energy Laboratory, 15013 Denver West Parkway, Golden, CO 80401 USA; 2 Department of Food Science, Cornell University, Ithaca, NY USA; 3 Biochemistry, Molecular and Cell Biology, Cornell University, Ithaca, NY USA

**Keywords:** GH48, Circular dichroism, X-ray crystallography, Cellulase, Molecular modeling

## Abstract

Glycoside hydrolase (GH) family 48 is an understudied and increasingly important exoglucanase family found in the majority of bacterial cellulase systems. Moreover, many thermophilic enzyme systems contain GH48 enzymes. Deletion of GH48 enzymes in these microorganisms results in drastic reduction in biomass deconstruction. Surprisingly, given their importance for these microorganisms, GH48s have intrinsically low cellulolytic activity but even in low ratios synergize greatly with GH9 endoglucanases. In this study, we explore the structural and enzymatic diversity of these enzymes across a wide range of temperature optima. We have crystallized one new GH48 module from *Bacillus pumilus* in a complex with cellobiose and cellohexaose (*Bpum*GH48). We compare this structure to other known GH48 enzymes in an attempt to understand GH48 structure/function relationships and draw general rules correlating amino acid sequences and secondary structures to thermostability in this GH family.

**Electronic supplementary material:**

The online version of this article (10.1186/s13068-017-0951-5) contains supplementary material, which is available to authorized users.

## Background

Enzymes in the glycoside hydrolase family 48 are believed to be an important member of many bacterial cellulase systems which also lack the better studied GH6 and GH7 family cellobiohydrolases produced by most cellulolytic fungi [1]. According to the CAZy database, family GH48 consists of 937 known enzymes, 903 of which are from bacterial organisms [2]. The most studied GH48 enzymes are GH48a from *Thermobifida fusca*, the cellulosomal GH48 CelS, and the non-cellulosomal GH48 CelY from *Clostridium thermocellum* [3–7]. Many GH48 enzymes are also produced by thermophilic and hyperthermophilic species, some of them being considered strong candidates for consolidated bioprocessing, making them attractive to study for potential applications in the nascent biofuels industry within thermal tolerant cellulase commercial preparations or consolidated Bioprocessing (CBP) applications. However, for a long time, there were only two protein structures deposited in the protein data bank (PDB) for this family of glycoside hydrolases making structure/function studies complicated. Recently, five more structures of family 48 glycoside hydrolases were deposited to the PDB, bringing the total number of known structures to seven. To broaden the structural database and directly correlate activity to structural features, we have characterized and expressed two GH48 domains from *C. bescii* and *B. pumilus* and solved their structures using X-ray crystallography. We have also determined the melting temperatures for these enzymes and determined their activity levels using phosphoric acid swollen cellulose (PASC) and bacterial microcrystalline cellulose (BMCC). We have selected five of these available PDB structures, used structural informatics and protein modeling approaches to identify features that may explain the observed thermostability variation in this family; as well the similar activity levels observed for these enzymes.

## Results and discussion

### Differences in stability but not in cellulolytic activity

**Fig. 1:**
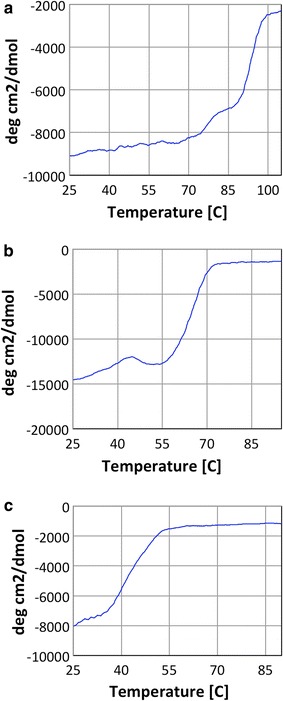
Circular Dichroism melt curves of three different GH48 catalytic domains. **a** *C. bescii*, **b** *T. fusca*, **c** *B. pumilus*

Circular dichroism spectroscopy was used to determine the melting temperature of each enzyme. The melting temperatures for purified GH48 enzymes isolated from *C. bescii*, *T. fusca*, and *B. pumilus*, were determined to be 80, 65, and 45 °C, respectively (Fig. 1). The wide range of melting temperatures for these constructs provides a range from thermophilic to mesophilic and it is interesting to note that the sequence identity for all these GH48 modules is high (between 46 and 62%) (see Table 1 and Additional file 1: Figure S1)—despite a 35 °C range in melting temperature for the enzymes. All the melting temperatures are as expected higher than the optimal growth temperatures of the host organism but not by a large margin.

**Table 1:** Characteristics of select glycoside hydrolase family 48 enzymes

PDB	Organism	Optimum growth temperature (°C)	*T*_*m*_ (°C)	Hphob_K&D_	% Sequence ID
4el8	*C. bescii*	75	80	140.4	100
1l1y	*C. thermocellum*	60	65^a^ [8]	141.7	62.7
4jjj	*T. fusca*	55	65	135.3	54.7
5cvy	*B. pumilus*	30	45	127.7	46.4
1f9d	*C. cellulolyticum*	34	37^a^ [9]	120.2	61.9

^a^Indicates optimal operating temperature

**Fig. 2:**
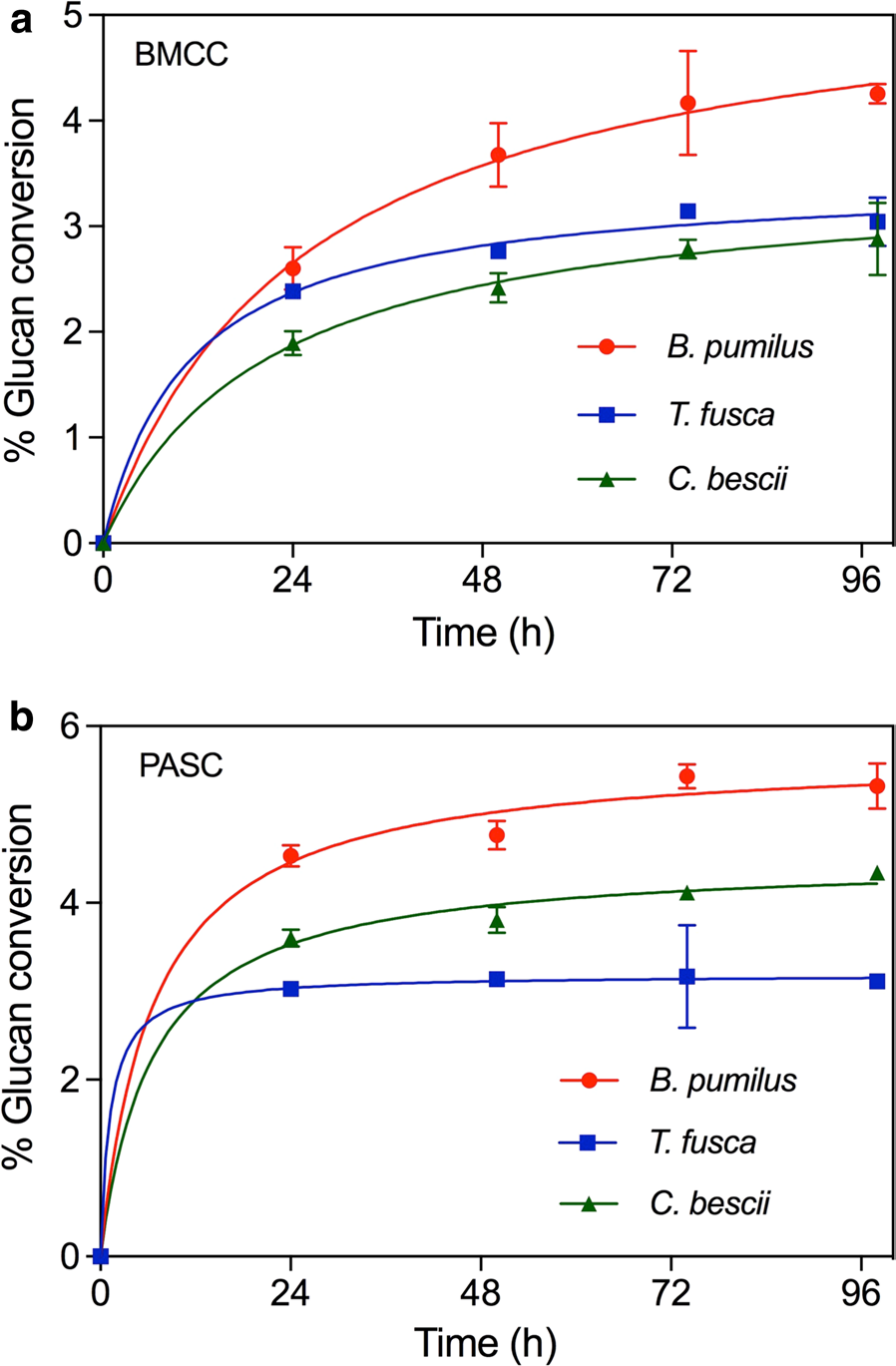
Glycoside Hydrolase family 48 enzymes display similar enzymatic activity on several substrates. **a** Glucan conversion of crystalline BMCC by three family 48 enzymes near their *T*_opt_, 75, 45, and 65 °C for *C. bescii* GH48, *B. pumilus* GH48, and *T. fusca* GH48, respectively. **b** Glucan conversion of low crystallinity PASC by three family 48 enzymes near their *T*_opt_, 75, 45, and 65 °C for *C. bescii* GH48, *B. pumilus GH48*, and *T. fusca*, respectively

The results of the enzyme digestions are shown in Fig. 2a, b. It is somewhat surprising that there is very little difference in the extent of conversion displayed by these enzymes considering the 35 °C difference in their temperature optima. The *B. pumilus* GH48 seems to outperform the other two GH48 enzymes by a small margin all at their optimal operating temperatures. Furthermore, it is also somewhat surprising that there is no apparent preference by these enzymes for neither the low crystallinity phosphoric acid swollen cellulose (PASC) substrate nor the more crystalline, bacterial microcrystalline cellulose (BMCC) substrate. It should be noted that low overall extents of conversion on insoluble substrates are typical for GH48 exoglucanases and can be improved with the addition of GH9 endoglucanases [7]. However, we would expect that the CelA GH48 from *C. bescii* would have the highest activity on cellulose based on both the high activity of this multi-modular enzyme and its much higher thermostability; whereas we would expect the *B. pumilus* GH48 to have significantly lower activity based on its much lower temperature optimum [10]. With regard to the *T. fusca* GH48, we would expect to find an intermediate level of activity based on its intermediate thermostability and previously reported activity measurements [7]. However, this result was not found in this study. When the enzymes are run at their temperature optima, we found that the *B. pumilus* GH48 seems slightly more active when compared to the other two enzymes under these conditions on both the crystalline and amorphous substrates tested (Fig. 2a, b).

### Can activity and thermostability be explained by direct structural comparisons?

Pair-wise secondary structure matching of structures with at least 70% secondary structure similarity by PDBeFold [11] found 36 unique structural matches for *Bpum*GH48 from the protein data bank. Most of these were different structures of CelS [12] or CelF [13]. After discarding different variants and mutants of the same protein, only six unique entries could be found. Out of these, CelF is most similar. *Bpum*GH48 has 51% sequence similarity and 77% secondary structure similarity with CelF (PDB code 1G9G). The C_α_ root mean square deviations of all compared structures varied between 0.91 and 1.02 Å showing that the overall backbone of all of the known GH48 structures is similar.

**Fig. 3:**
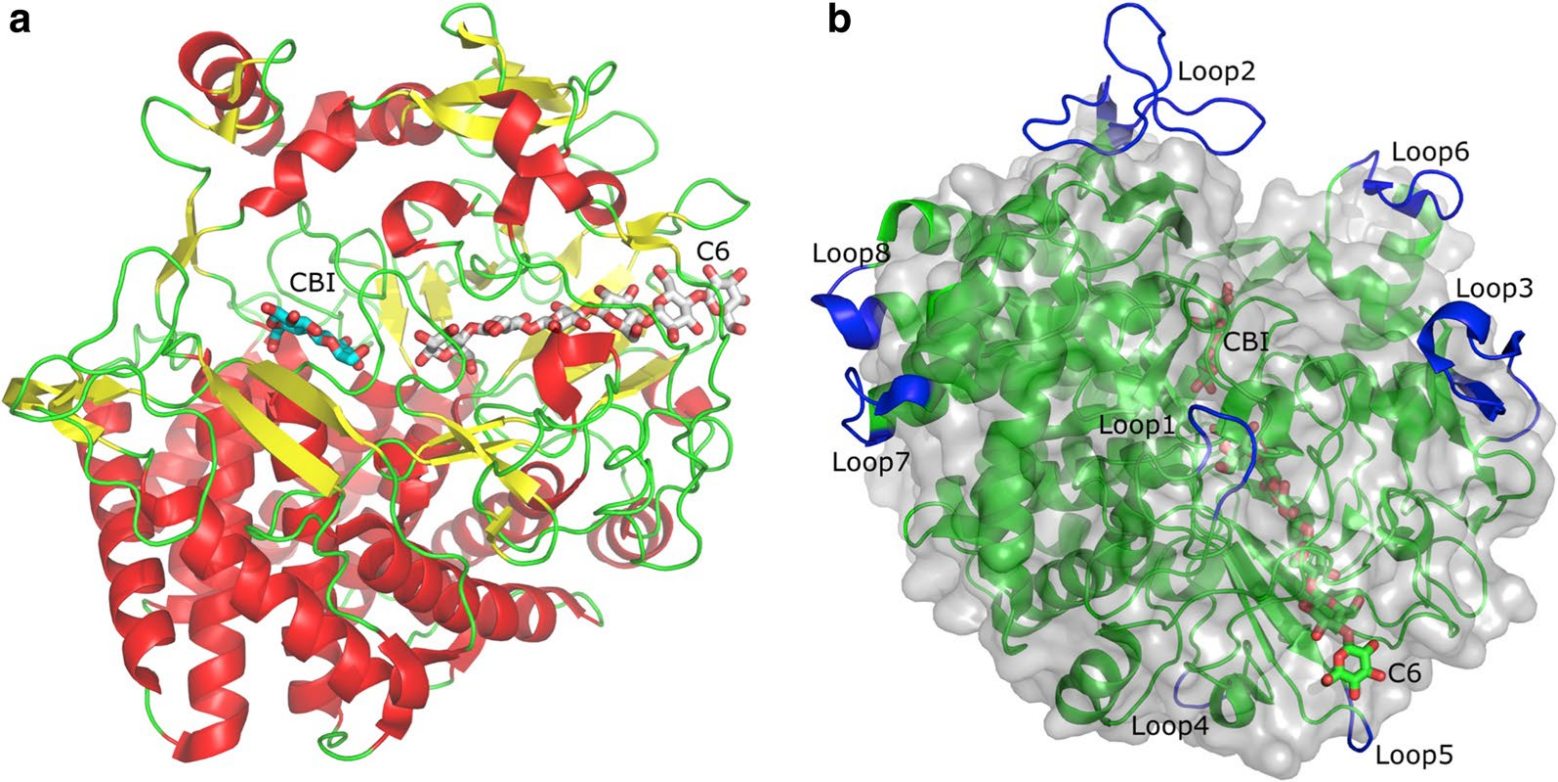
*Bpum*GH48 Structure. **a** Cartoon representation of *Bpum*GH48 structure with cellobiose and cellohexaose. α-helices are shown in red, β-strands in yellow and loops in green. The bound cellobiose molecule (CBI) is depicted as sticks with cyan carbons and red oxygens and the cellohexaose substrate (C6) is shown as sticks with gray carbons and red oxygens. **b** Comparison of surface loops between *Bpum*GH48 and CelF GH48 (PDB code 1G9G). The overall structure of *Bpum*GH48 is visualized using a green cartoon representation and the unusually large loops are colored blue. The cellobiose and cellohexaose molecules are shown as sticks with green carbons and red oxygen atoms. For CelF GH48 only a transparent surface is shown to highlight the differences. The unusually large loops of *Bpum*GH48 are labeled from 1 to 8 to help with discussion

All GH48 domains have (*α*/*α*)_6_ barrel fold (Fig. 3a) with nearly identical organization of the active site tunnel. The tunnel layout is depicted in great details in [6] and [10]. What is remarkable is the conservation of the tunnel structure throughout the five GH48s considered in this study. Out of 36 residues that represent the tunnel walls and contact with the substrate/product, 27 are universally conserved and most of the rest are highly conserved (Table 2). We believe that this highly conserved substrate-binding tunnel is the main explanation for the similar levels of activity shown by different GH48s.

**Table 2:** Highly conserved tunnel-forming residues of GH48s

*B. pumilus*	*C. bescii*	*T. fusca*	*C. thermocellum*	*C. cellulolyticum*
His30	His	His	His	His
Glu38	Glu	Glu	Glu	Glu
Ala39	Ala	Ala	Ala	Ala
Glu49	Glu	Glu	Glu	Glu
Thr106	Thr	Thr	Thr	Thr
Ala108	Ala	**Ile**	Ala	Ala
Leu121	Leu	Leu	Leu	Leu
Trp150	Trp	Trp	Trp	Trp
Asn174	Asn	Asn	Asn	Asn
Phe176	Phe	**Tyr**	Phe	Phe
Gln177	Gln	Gln	Gln	Gln
Thr210	**Ile**	Thr	Thr	Thr
**Glu212**	Asp	Asp	Asp	Asp
**Gln214**	Asn	Asn	**Ser**	**Gly**
Gln219	Gln	Gln	Gln	Gln
Arg221	Arg	Arg	Arg	**Lys**
Thr223	Thr	Thr	Thr	Thr
Asp227	Asp	Asp	Asp	Asp
Lys267	Lys	Lys	Lys	Lys
Tyr268	Tyr	Tyr	Tyr	Tyr
Trp295	Trp	Trp	Trp	Trp
Tyr296	Tyr	Tyr	Tyr	Tyr
**Ser298**	Ala	**Ser**	Ala	Ala
Trp309	Trp	Trp	Trp	Trp
Trp311	Trp	Trp	Trp	Trp
Ile313	Ile	Ile	Ile	Ile
Tyr322	Tyr	Tyr	Tyr	Tyr
Tyr403	Tyr	**Trp**	Tyr	Tyr
Trp411	Trp	Trp	Trp	Trp
**Met414**	Phe	Phe	Phe	**Met**
Trp417	Trp	Trp	Trp	Trp
Asp534	Asp	Asp	Asp	Asp
Glu590	Glu	Glu	Glu	Glu
Arg592	Arg	**Pro**	Arg	Arg
Arg682	Arg	Arg	Arg	Arg
Trp684	Trp	Trp	Trp	Trp

Non-conserved residues are shown in bold font

The most unique feature about the structure of *Bpum*GH48 is the eight unusually long peptide inserts that result in extra loops on the surface of the molecule when compared to other GH48 enzymes (Fig. 3b). Beyond these loops, the core structure is very similar to other well described GH48 enzymes, such as CelF GH48 [13], CelS GH48 [12], or *T. fusca* GH48 [3]. Compared to CelF GH48, *Bpum*GH48 has 80 more residues between Ser2 and Leu702 when the two structures are superimposed. Loop 5 (Leu303 to Asn308) is located near the tunnel entrance and loops 2 (Arg463 to Ala493) and 6 (Phe664 to Gly674) are near the exit. Due to their position, these loops have the potential to affect substrate-binding and product expulsion. Although, computer simulations indicate that the *Bpum*GH48 loops near the tunnel exit do not affect product inhibition [5]. However, they could be one of the reasons for the lower thermostability of *Bpum*GH48 by being more exposed to solvent. Without extensive mutational experiments, we are not able to deduce a clear role for them.

### More detailed comparisons are necessary to explain thermostability and potentially activity

**Fig. 4:**
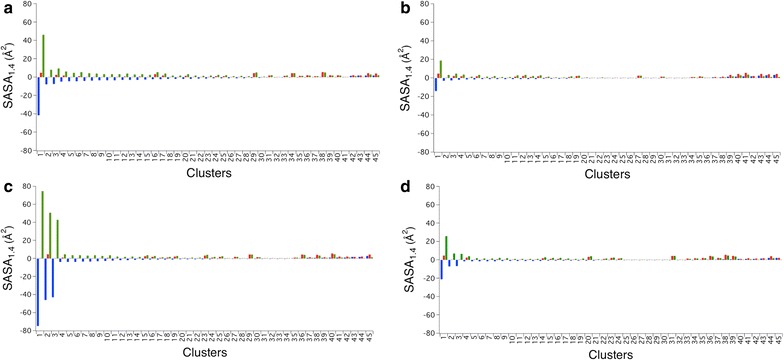
Glycoside Hydrolase family 48 enzymes display similar atomic packing compared to mesophilic enzyme clusters. SASA_1.4_ values for residue clusters from the GH48 structure pairs are shown, with the more thermostable enzyme clusters shown in red, the less thermostable enzyme clusters in green and the difference, ΔSASA_1.4_, in blue. Values are sorted by ΔSASA_1.4_. **a** SASA_1.4_ values are shown comparing clusters from the *C. bescii* (PDB 4el8) and *C. thermocellum* (PDB 1l1y) GH48 enzymes, which have a difference in melting temperature of 15 °C, **b** the *C. bescii* and *T. fusca* (4jjj) GH48 enzymes, which have a difference in melting temperature of 15 °C., **c** the *C. bescii* and *B. pumilus* (PDB 5bv9) GH48 enzymes, with a difference in melting temperature of 35 °C, **d** and the *C. bescii* and *C. cellulolyticum* (PDB 1f9d) GH48 enzymes, with a difference in melting temperature of 43 °C

The GH48 enzymes evaluated here represent a wide range of thermostability within this family, covering melting temperatures from 37 to 80 °C. More detailed comparisons of the X-ray crystal structures allow us to evaluate and compare these GH48 family members to identify features contributing to differences in stability or activity. Previous research from our group comparing mesophilic and thermophilic enzymes determined that the quality of amino acid side-chain packing is often improved in thermophilic enzymes compared to mesophilic homologues [14]. Even though this work suggests thermophilic enzymes cannot tolerate imperfections, such as poor side-chain packing, some mesophilic homologues display similarly optimized side-chain packing suggesting alternative mechanisms must be responsible for differences in thermostability. Here, we applied the same analysis, comparing the atomic packing, or side-chain packing, for clusters of interacting residues throughout the core of the proteins. We compared the most thermostable family member, the GH48 from *C. bescii*, to each of the other family members. A negative ΔSASA_1.4_ (Solvent Accessible Surface Area) indicates the *C. bescii* enzyme cluster displays smaller and/or fewer cavities, demonstrating improved atomic packing compared to the corresponding cluster from the other GH48 family member. We find that among the 5 GH48 enzymes all display comparable and optimized side-chain packing (Fig. 4).

**Fig. 5:**
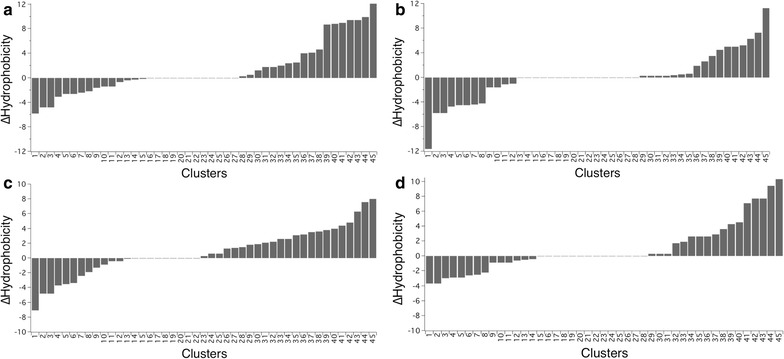
More thermostable Glycoside Hydrolase family 48 enzymes display more hydrophobicity in residue clusters compared to mesophilic enzyme clusters. Hydrophobicity scores (Hphob_K&D_) for residue clusters from the GH48 structure pairs are shown. Values are sorted by Hphob_K&D_. **a** Hphob_K&D_ values are shown comparing clusters from the *C. bescii* (PDB 4el8) and *C. thermocellum* (PDB 1l1y) GH48 enzymes, which have a difference in melting temperature of 15 °C, **b** the *C. bescii* and *T. fusca* (4jjj) GH48 enzymes, which have a difference in melting temperature of 15 °C., **c** the *C. bescii* and *B. pumilus* (PDB 5bv9) GH48 enzymes, with a difference in melting temperature of 35 °C, **d** and the *C. bescii* and *C. cellulolyticum* (PDB 1f9d) GH48 enzymes, with a difference in melting temperature of 43 °C

The hydrophobic effect drives protein folding and hydrophobic interactions often contribute significantly to protein binding affinity [15]. Removing a buried methylene or methyl group can destabilize a protein, with examples showing destabilization of more than 1 kcal/mol [15, 16]. Alternatively, introducing new methylene groups can stabilize a protein, presenting a mechanism that protein design algorithms have used to rationally increase the thermostability by identifying positions that can accommodate larger hydrophobic amino acids [13, 15, 16, 27]. Here, we compare the hydrophobicity of the protein core regions for each GH48 family member from Table 1. Scores comparing the hydrophobicity of the twenty amino acids were developed based on the idea that protein unfolding would transfer hydrophobic residues to the aqueous solvent environment, a process which is energetically unfavorable [17]. Using the amino acid hydrophobicity scale of Kyte and Doolittle, we see that the hydrophobic score (Hphob_K&D_ in Table 1) correlates with the *T*_*m*_ of each GH48 enzyme (Table 1) with more thermostable GH48 family members having higher Hphob_K&D_ scores [17]. The total hydrophobicity scores for each GH48 family member can change and may not be representative of overall stability. Therefore, we conducted a more detailed analysis by comparing the hydrophobicity for each cluster of interacting residues, as was done to compare differences in side-chain packing (ΔSASA_1.4_) in Fig. 4. We compared the most thermostable family member, the GH48 from *C. bescii*, to each of the other family members. ΔHydrophobicity represents the differences between the Hphob_K&D_ scores for each of the *C. bescii* GH48 residue clusters across GH48 family members. Positive ΔHydrophobicity values represent *C. bescii* clusters that are more hydrophobic compared to the corresponding clusters in other family members. *C. bescii* GH48 has a great number of clusters that are more hydrophobic, indicating increased buried hydrophobicity, which may explain the difference in stability between the *C. bescii* GH48 and other less thermostable family members (Fig. 5).

When considering the overall activity of these proteins, given that the catalytic residues are identical and the catalytic tunnel residues are highly conserved, we have examined the SASA, which may explain what we believe to be responsible for their similar activity. We note that all of the structures are uniformly well packed, mostly equally across the family members that we evaluated. This result indicates that there should be an equivalent freedom of motion within the core of each enzyme and this could explain the lack of kinetic differences between these enzymes, as we have observed roughly similar activities for them.

## Conclusions

We have utilized classical biochemistry approaches to study three different family 48 glycoside hydrolases, which display widely different temperature optima. Additionally, we used X-ray crystallography and computational analyses to explain the difference (or lack thereof) between cellulolytic activity and thermostability. To summarize, we have demonstrated that the three GH48 exoglucanases tested have very different melting temperatures despite having high sequence identity and similar enzymatic activity. Based on sequence and structural alignments as well as the molecular modeling, we conclude that some of these differences lay in the loop regions of these proteins but also in differences in the hydrophobic clusters within the proteins. If these explanations are correct, we may be able to modify the temperature optima of GH48 exoglucanases in the future. Additionally, they may be examples of how thermostability is modulated in other enzymes.

## Methods

### Cloning, overexpression, and purification of CelA CBM3-GH48 isolated from *C. bescii*

Cloning, overexpression, and purification of CelA CBM3-GH48 isolated from *C. bescii*: PCR fragment of CBM3-GH48 was make by two primers F-CBM3v-*Nhe*I of ACACCGGCTAGCAGCAGCACACCTGTAGCAGG and R-GH48-*Xho*I of TAGCTTCTCGAGTTATTGATTGCCAAACAGTA, and its template was the *C. bescii* genomic DNA. The PCR fragment was inserted into pET28a with *Nhe*I and *Xho*I. The correct insert was verified by DNA sequencing. Plasmid with the target gene was overexpressed in *E. coli* BL21 (DE3) strain (Ipswich, MA, USA). The gene expression was induced by 0.3 IPTG under 16 °C. The cells was harvested and lysed by sonication, and was purified by Nickel-NTA (Invitrogen, Grand Island, NY, USA). The affinity purified protein was further purified using hydrophobic interaction chromatography using a Source 15 phenyl resin column (GE) and 20 mM Acetate ph5 1 M ammonium sulfate buffer followed by size exclusion chromatography (SEC) using a Superdex 75 column using 20 mM Acetate pH5 100 mM NaCl.

### Cloning, overexpression, and purification of *B. pumilus* GH48

The *B. pumilus* GH48 construct was synthesized and codon optimized for *E. coli* expression and placed in a pMal (NEB) MBP expression vector with a Genenase cleavage site. The plasmid with the target gene was overexpressed in *E. coli* BL-21(DE3), induced with 0.3 mM IPTG and induced for 21 h at 17 °C. Cells were pelleted at 10,000×*g* and re-suspended in 40 mL Bugbuster (EDM Millipore) with C-Complete protease inhibitor (Sigma) and then sonicated for one min and allowed to incubate for at RT for one h. Cell debris was then pelleted by centrifugation at 10,000×*g* and the remaining supernatant was added to buffer-equilibrated amylose beads and incubated for one h and then washed with 20 mM Tris buffer pH 7.4 with 200 mM NaCL. The protein was then released from the beads with Genenase I.

The resultant mix of maltose binding protein (MBP), MBP fusion, and cleaved GH48 then was then separated by SEC to separate the MBP and then further purified using anion exchange chromatography (AEC) with a Source 15Q column pH 6.8 Tris buffer with 2 M NaCl. Finally, hydrophobic interaction chromatography (HIC) using a Source 15-phe column (GE) with 20 mM pH 5.0 acetate buffer and a 1 M ammonium sulfate gradient.

### *T. fusca* GH48

*T. fusca* GH48 was provided by David Wilson’s laboratory and produced as described in [7].

### Circular dichroism (CD)

CD measurements were carried out using a Jasco J-715 spectropolarimeter with a jacketed quartz cell with a 1.0 mm path length. The cell temperature was controlled to within ±0.1 °C by circulating 90% ethylene glycol using a Neslab R-111 m water bath (NESLAB Instruments, Portsmouth, NH, U.S.A.) through the CD cell jacket. The results were expressed as mean residue ellipticity [*è*]_mrw_. The spectra obtained were averages of five scans. The spectra were smoothed using an internal algorithm in the Jasco software package, J-715 for Windows. Protein samples were studied in 20 mM sodium acetate buffer, pH 5.0 with 100 mM NaCl at a protein concentration of 0.35 mg/mL for the near UV CD. Thermal denaturation of different constructs was monitored by CD in the near UV (190–260 nm) region. For the analysis of thermostability, the temperature was increased from 55 to 105 °C with a step size of 0.2 °C, and monitored at a wavelength of 222 nm.

### Enzyme digestions

The GH48 enzymes were loaded at a concentration of 20 mg protein per g glucan to 1.5% w/w solutions of phosphoric acid swollen cellulose (PASC) and bacterial microcrystalline cellulose (BMCC). Bacterial microcrystalline cellulose (BMCC) was prepared from BC as described previously [18]. Assays were carried out at 75, 60, and 37 °C in 20 mM acetate buffer, pH 5.5 containing 10 mM CaCl_2_, and 100 mM NaCl. Digestion assays were performed in triplicate, and the final glucose concentration was determined using HPLC. To measure cellulose conversion, 60 μL of each hydrolysate sample was diluted tenfold and filtered using a 0.45 µm filter. Glucose concentrations were measured by HPLC (Agilent) using an Aminex HPX-87H column (BioRad Laboratories) using a 5 mM sulfuric acid mobile phase and a flow rate of 0.6 mL/min. The sample injection volume was 20 µL and the run time was 11 min. For sugar product determination, digestion aliquots were analyzed using an ICS-5000+ System (Thermofisher Scientific) equipped with a Carbopac PA20 column/guard column and pulsed amperometric detection (PAD). Monomeric sugars and xylobiose were eluted at 0.45 mL/min using an isocratic eluent concentration of 32.5 mM NaOH. The carbohydrate (quad potential) waveform for an Ag/AgCl reference electrode was used for detection and quantitation. The glucose concentration from each reaction was divided by the maximal glucose yield obtained from compositional analysis, in order to calculate a fractional glucan conversion for each reaction.

### Crystallization

*Bpum*GH48 crystals in complex with cellobiose (*Bpum*GH48-C2) and cellobiose/cellohexaose (*Bpum*GH48-C2C6) were initially obtained with sitting drop vapor diffusion using a 96-well plate with Grid Screen Salt HT from Hampton Research (Aliso Viejo, CA). 50 µL of well solution was added to the reservoir and drops were made with 0.2 µL of well solution and 0.2 µL of protein solution using a Phoenix crystallization robot (Art Robbins Instruments, Sunnyvale, CA). The crystals were grown at 20 °C using screens containing 1–3 M malonate with pH 5–7 and 20 mM cellobiose. The protein solutions contained 15 mg/mL of protein in 20 mM acetate buffer pH 5, with 100 mM NaCl and 10 mM CaCl_2_. Before freezing crystals were briefly soaked in a drop containing excess amounts of cellohexaose, 10% (v/v) glycerol and 10% (v/v) ethylene glycol.

### Data collection and processing

The *Bpum*GH48 crystals were flash frozen in a nitrogen gas stream at 100 K before home source data collection using an in-house Bruker X8 MicroStar X-Ray generator with Helios mirrors and Bruker Platinum 135 CCD detector. Data were indexed and processed with the Bruker Suite of programs version 2014.9 (Bruker AXS, Madison, WI).

### Structure solution and refinement

Intensities were converted into structure factors and 5% of the reflections were flagged for R_free_ calculations using programs F2MTZ, Truncate, CAD, and Unique from the CCP4 package of programs [19]. The program MOLREP [20] version 11.2.08 was used for molecular replacement using the unliganded structure of a family 48 glycoside hydrolase from *C. bescii* (PDB entry 4EL8 [10]) as the search model. Refinement and manual correction was performed using REFMAC5 [21] version 5.8.135 and Coot [22] version 0.8.2. The MOLPROBITY method [23] was used to analyze the Ramachandran plot and root mean square deviations (rmsd) of bond lengths and angles were calculated from ideal values of Engh and Huber stereo chemical parameters [24]. Wilson B-factor was calculated using CTRUNCATE version 1.15.10 [19]. The data collection and refinement statistics are shown in Table 1.

The structure of *Bpum*GH48-C2C6 with cellobiose and cellohexaose was refined to a resolution of 2.0 Å with *R* and *R*_free_ of 0.146 and 0.189, respectively. There is one molecule in the asymmetric unit with a cellobiose and a cellohexaose molecule (Fig. 3). It has an (alpha/alpha)_6_ barrel fold with several malonate, ethylene glycol, and glycerol molecules on the surface. This structure has been deposited in the Protein Data Bank (PDB) with code 5CVY. The structure of *Bpum*GH48-C2 with cellobiose (PDB code 5BV9) was solved at resolution 1.93 Å and *R* and *R*_free_ of 0.161 and 0.207, respectively. X-ray data collection and refinement statistics and details are listed in Additional file 1: Table S1.

### Structure analysis

Programs Coot [22], PyMOL (http://www.pymol.org) and ICM (http://www.molsoft.com) were used for comparing and analyzing structures. Figure 3 was created using PyMOL.

### Protein sequence analysis

Sequences were aligned and analyzed using the MacVector software (MacVector, Inc., Cary, NC) [25]. Sequence alignments were performed using the GONNET substitution matrix [26], with a gap opening penalty of 10 and a gap extension penalty of 0.05.

### Identification of residue clusters

Residue clusters were determined as previously described [14]. Briefly, interacting residues were identified using a distance cutoff of 3 Å between side-chain heavy atoms (C, N, O and S) using the protein design software, Rosetta [27, 28]. Structurally equivalent residue clusters in homologous mesophilic enzymes were identified using the structural alignment algorithm, jFatCat flexible [29]. Residue clusters were filtered based on degree of solvent accessibility, selecting only clusters where each residue displayed less than 3 Å^2^ of SASA as determined using Naccess [30].

### Comparing structurally equivalent residue clusters

The residue accessible surface areas were computed using the program Naccess. Naccess rolls a probe of a given radius over the van der Waals surface of a molecule to trace the accessible surface. A probe of radius 1.4 Å was used here to reflect the radius of water and thus the solvent accessible surface area. Graphs were generated using IGOR Pro (WaveMetrics Inc., Lake Oswego, OR).

## Additional file

**Additional file 1.** Additional table and figure.
