# Supplementary material for: Natural diversity of glycoside hydrolase family 48 exoglucanases: insights from structure
Source: Biotechnol Biofuels. 2017 Nov 30;10:274. doi: 10.1186/s13068-017-0951-5 (PMC5707792; doi:10.1186/s13068-017-0951-5)
Supplement: Supplementary file 1 — Additional file 1. Additional table and figure. [file 13068_2017_951_MOESM1_ESM.docx]

**Table S1**. X-ray data collection and refinement statistics. Statistics for the highest resolution bin are in parenthesis.

|  | | *Bpum*GH48-C2C6 | *Bpum*GH48-C2 |
| --- | --- | --- | --- |
| **Data Collection** |  |  |  |
| Space group | | P4_3_2_1_2 | P4_3_2_1_2 |
| Unit cell, Å, ° | | a= b= 98.04, c = 218.96  α= β= γ= 90.0 | a= b= 97.29, c = 218.11  α= β= γ = 90.0 |
| Wavelength, Å | | 1.54188 | 1.54188 |
| Temperature (K) | | 100 | 100 |
| Resolution, Å | | 25.0-2.0 (2.1–2.0) | 25.0-1.93 (2.03-1.93) |
| Unique reflections | | 72223 (9136) | 79431 (11018) |
| R_int_^†^ | | 0.123 (0.438) | 0.171 (0.529) |
| Average redundancy | | 7.6 (4.2) | 8.7 (3.9) |
| <I>/<σ(I)> | | 14.8 (3.6) | 9.6 (2.0) |
| Completeness, % | | 99.0 (93.8) | 99.8 (99.9) |
| **Refinement** | |  |  |
| Resolution, Å | | 25-2.0 (2.05-2.0) | 25-1.93 (1.98-9.93) |
| R/R_free_ | | 0.146 (0.189) / 0.228 (0.246) | 0.161 (0.299) / 0.207 (0.308) |
| Protein atoms | | 5816 | 5692 |
| Water molecules | | 831 | 1037 |
| Other atoms | | 219 | 100 |
| RMSD from ideal bond length, Å^#^ | | 0.019 | 0.020 |
| RMSD from ideal bond angles, ° ^#^ | | 1.849 | 1.912 |
| Wilson B-factor | | 9.4 | 14.8 |
| Average B-factor for protein atoms, Å^2^ | | 15.7 | 16.5 |
| Average B-factor for water molecules, Å^2^ | | 25.8 | 26.1 |
| Ramachandran plot statistics, %* | |  |  |
| Allowed | | 99.7 | 99.5 |
| Favored | | 97.2 | 97.3 |
| Outliers | | 2 (Ala39, Asp88) | 4 (Glu38, Ala39, Asp88, Gly89) |

† Rint = ∑| I - <I> | / ∑|I| where I is the intensity of an individual reflection and <I> is the mean intensity of a group of equivalents and the sums are calculated over all reflections with more than one equivalent measured

# R. A. Engh, R. Huber, Accurate Bond and Angle Parameters for X-Ray Protein-Structure Refinement. *Acta Crystallographica Section A* **47**, 392-400 (1991).

* Chen, V. B., Arendall, W. B., 3rd, Headd, J. J., Keedy, D. A., Immormino, R. M., Kapral, G. J., Murray, L. W., Richardson, J. S. & Richardson, D. C. (2010). *Acta Crystallographica Section D* **66**, 12-21.

**Figure S1**

10 20 30 40 50 60

4el8 ---------G-EYGQRFMWLWNKIHDPANGYFNQD-G--IPYHSVETLICEAPDYGHLTTSEAFS

1l1y GPTKAPTKDGTSYKDLFLELYGKIKDPKNGYFSPDEG--IPYHSIETLIVEAPDYGHVTTSEAFS

4jjj -----------SYDQAFLEQYEKIKDPASGYFREFNGLLVPYHSVETMIVEAPDHGHQTTSEAFS

5cvy -----------SNKERFLTLYHQIKSDANGYFSPE-G--IPYHSIETLICEAPDYGHMTTSEAYS

1f9d ----ASSPANKVYQDRFESMYSKIKDPANGYFSEQ-G--IPYHSIETLMVEAPDYGHVTTSQAMS

70 80 90 100 110 120 130

4el8 YYVWLEAVYGKLTGDWSKFKTAWDTLEKYMIPSAE----DQP---MS-YDPNKPATYAGEWETPD

1l1y YYVWLEAMYGNLTGNWSGVETAWKVMEDWIIPDST----EQPG--MSSYNPNSPATYADEYEDPS

4jjj YYLWLEAYYGRVTGDWKPLHDAWESMETFIIPGTK----DQPT--NSAYNPNSPATYIPEQPNAD

5cvy YWLWLEVLYGHYTRDWSKLEAAWDNMEKYIIPVNEDGNDEQPH--MSAYNPSSPATYASEKPYPD

1f9d YYMWLEAMHGRFSGDFTGFDKSWSVTEQYLIPTEK----DQPNTSMSRYDANKPATYAPEFQDPS

140 150 160 170 180 190

4el8 KYPSPLEFN-VPVGKDPLHNELVSTYGSTLMYGMHWLMDVDNWYGYGKRGDGV-SRASFINTFQR

1l1y YYPSELKFDTVRVGSDPVHNDLVSAYGPN-MYLMHWLMDVDNWYGFG-TG----TRATFINTFQR

4jjj GYPSPLMNN-VPVGQDPLAQELSSTYGTNEIYGMHWLLDVDNVYGFGFCGDGTDDAPAYINTYQR

5cvy QYPSQLSGA-RPAGQDPIDGELKSTYGTNETYLMHWLLDVDNWYKYGNLLNPS-HKAAYVNTFQR

1f9d KYPSPLDTS-QPVGRDPINSQLTSAYGTSMLYGMHWILDVDNWYGFGARADGT-SKPSYINTFQR

200 210 220 230 240 250 260

4el8 GPEESVWETVPHPSWEEFKWGGPN-GFLDLFIKDQ-NYSKQWRYTDAPDADARAIQATYWAKVWA

1l1y GEQESTWETIPHPSIEEFKYGGPN-GFLDLFTKDR-SYAKQWRYTNAPDAEGRAIQAVYWANKWA

4jjj GARESVWETIPHPSCDDFTHGGPN-GYLDLFTDDQ-NYAKQWRYTNAPNADARAVQVMFWAHEWA

5cvy GQQESVWEAIPHPSQDDKSFGKPNEGFMSLFTKENQVPAAQWRYTNATDADARAIQAIYWA----

1f9d GEQESTWETIPQPCWDEHKFGGQY-GFLDLFTKDTGTPAKQFKYTNAPDADARAVQATYWADQWA

270 280 290 300 310 320

4el8 KEQGKFNEISSYVAKAAKMGDYLRYAMFDKYFKPLGC---QDKNAAGGTGYDSAHYLLSWYYAWG

1l1y KEQGKGSAVASVVSKAAKMGDFLRNDMFDKYFMKIGA---QDKTPA--TGYDSAHYLMAWYTAWG

4jjj KEQGKENEIAGLMDKASKMGDYLRYAMFDKYFKKIGNCVGATSCPGG-QGKDSAHYLLSWYYSWG

5cvy KELGY--NNSTYLDKAKKMGDFLRYGMYDKYFQTIGSGKQGNPYPG--NGKGACHYLMAWYTSWG

1f9d KEQGK--SVSTSVGKATKMGDYLRYSFFDKYFRKIG----QPSQAG--TGYDAAHYLLSWYYAWG

330 340 350 360 370 380 390

4el8 G-----ALWSWKIGSSHVHFGYQNPMAAWALAND-SDMKPKSPNGASDWAKSLKRQIEFYRWLQS

1l1y GGIG--ASWAWKIGCSHAHFGYQNPFQGWVSATQ-SDFAPKSSNGKRDWTTSYKRQLEFYQWLQS

4jjj GSLD--SAWAWRIGSSSSHQGYQNVLAAYALSQV-PELQPDSPTGVQDWATSFDRQLEFLQWLQS

5cvy GGLGDYANWSWRIGASHCHQGYQNPVAAYALSSDKGGLKPSSATGASDWEKTLKRQLEFYVWLQS

1f9d GGID--STWSWIIGSSHNHFGYQNPFAAWVLSTD-ANFKPKSSNGASDWAKSLDRQLEFYQWLQS

400 410 420 430 440 450

4el8 AEGAIAGGATNSWNGRYEKYPAGTATFYGMAYEPNPVYHDPGSNTWFGFQAWSMQRVAEYYY---

1l1y AEGGIAGGATNSWNGRYEKYPAGTSTFYGMAYVPHPVYADPGSNQWFGFQAWSMQRVMEYYL---

4jjj AEGGIAGGATNSWKGSYDTPPTGLSQFYGMYYDWQPVWNDPPSNNWFGFQVWNMERVAQLYY---

5cvy KEGAIAGGATNSWNGDYSAYPAGRSTFYDMAYEDAPVYHDPPSNNWFGMQAWPMERVAELYYIFV

1f9d AEGAIAGGATNSWNGRYEAVPSGTSTFYGMGYVENPVYADPGSNTWFGMQVWSMQRVAELYY---

460 470 480 490 500 510 520

4el8 VTGDKD------AGALLEKWVSWVKSVVKL------------NSD-G------------------

1l1y ETGDSS------VKNLIKKWVDWVMSEIKL------------YDD-G------------------

4jjj VTGDAR------AEAILDKWVPWAIQHTDV------------DADNGG----------------Q

5cvy KDGDKTSENVQMAKSCITKWVNYALDYIFIGSRPVSDEEGYFLDDQGRRILGGTNATVATTSAPG

1f9d KTGDAR------AKKLLDKWAKWINGEIKF------------NAD-G------------------

530 540 550 560 570 580

4el8 TFAIPSTLDWSGQPDTWNGA--YTGNSNLHVKVVDYGTDLGITASLANALLYYSAGTKKY-G---

1l1y TFAIPSDLEWSGQPDTWTGT--YTGNPNLHVRVTSYGTDLGVAGSLANALATYAAATERWEG---

4jjj NFQVPSDLEWSGQPDTWTGT--YTGNPNLHVQVVSYSQDVGVTAALAKTLMYYAKRSG-------

5cvy EFWLPGNIAWSGQPDTWNGFQSATGNPNLTAVTKDPTQDTGVLGSLVKAFTFFAAATKLETGNYT

1f9d TFQIPSTIDWEGQPDTWNPTQGYTGNANLHVKVVNYGTDLGCASSLANTLTYYAAKSG-------

590 600 610 620 630 640 650

4el8 VFDEGAKNLAKELLDRMW-KLYRDE-KGLSAPEK----RADYKRFFEQEVYIPAGWIGKMPNGDV

1l1y KLDTKARDMAAELVNRAWYNFYCSEGKGVVTEEA----RADYKRFFEQEVYVPAGWSGTMPNGDK

4jjj --DTTALATAEGLLDALL--AHRDS-IGIATPEQPSWDRLDDPWDGSEGLYVPPGWSGTMPNGDR

5cvy ALGVRAKDAAAQLLEVAW--NYNDG-VGIVTEEE----REDYDRFFKKEVYFPNGWNGTFGQGNQ

1f9d --DETSRQNAQKLLDAMW-NNYSDS-KGISTVEQ----RGDYHRFLDQEVFVPAGWTGKMPNGDV

660 670 680 690 700 710

4el8 I----------KSG-----VKFIDIRSKYKQDPDWPKLEAAYK------SGQ----APEFRYHRF

1l1y I----------QPG-----IKFIDIRTKYRQDPYYDIVYQAYL------RGE----APVLNYHRF

4jjj I----------EPG-----ATFLSIRSFYKNDPLWPQVEAHLN------DPQNVP-APIVERHRF

5cvy IPGSSTIPSDPQRGGNGVYTSFADLRPNIKQDPAWSSLESKYQSSFNEATGKWENGAPVFTYHRF

1f9d I----------KSG-----VKFIDIRSKYKQDPEWQTMVAALQ------AGQ----VPTQRLHRF

720 730 740

4el8 WAQCDIAIAN---ATY--EILF---

1l1y WHEVDLAVAMGVLATYFPDMTYKVP

4jjj WAQVEIATAF---AAH--DELFG--

5cvy WSQVDMATAY---AEY--HRLINL-

1f9d WAQSEFAVAN---GVY--AILFPD-
